# Clinical and biochemical investigation of male patients exhibiting membranous cytoplasmic bodies in biopsied kidney tissues; a pitfall in diagnosis of Fabry disease

**DOI:** 10.12860/jnp.2015.17

**Published:** 2015-07-01

**Authors:** Hitoshi Sakuraba, Takahiro Tsukimura, Toshie Tanaka, Tadayasu Togawa, Naoki Takahashi, Daisuke Mikami, Sachiko Wakai, Yasuhiro Akai

**Affiliations:** ^1^Department of Clinical Genetics, Meiji Pharmaceutical University, Kiyose, Tokyo, Japan; ^2^Departmentof Functional Bioanalysis, Meiji Pharmaceutical University, Kiyose, Tokyo, Japan; ^3^Division of Nephrology, Department of General Medicine, Faculty of Medical Sciences, University of Fukui, Eiheiji, Fukui, Japan; ^4^Division of Nephrology, Department of Internal Medicine, Ohkubo Hospital, Tokyo Metropolitan Health and Medical Treatment Corporation, Shinjuku, Tokyo, Japan; ^5^The 1st Department of Internal Medicine, Nara Medical University, Kashihara, Nara, Japan

**Keywords:** Fabry disease, Inclusion body, Acquired lysosomal disease, α-Galactosidase A, Globotriaosylsphingosine

## Abstract

*Background:* The existence of membranous cytoplasmic bodies in biopsied kidney tissues is one of the important findings when considering Fabry disease as the first choice diagnosis. However, there are possible acquired lysosomal diseases associated with pharmacological toxicity, although less attention has been paid to them.

*Case Presentation:* We experienced 3 male patients presenting with proteinuria and specific pathological changes strongly suggesting Fabry disease. We sought detailed clinical and biochemical information to avoid a wrong diagnosis. The patients were examined clinically and pathologically, and plasma α-galactosidase A (GLA) activity and the globotriaosylsphingosine (lyso-Gb3) concentrations were measured. Electron microscopic examination revealed numerous membranous inclusion bodies in podocytes, and biochemical analysis revealed normal GLA activity and a normal lyso-Gb3 level in plasma, showing that they did not have Fabry disease. They suffered from hyperlipidemia, myeloma, or lupus nephritis. They had received pitavastatin calcium, clarithromycin, loxoprofen and/or prednisolone, and there was no medication history of cationic amphiphilic drugs.

*Conclusions:* In this case series, the etiology of the inclusions was not clarified. However, these cases indicate that careful attention should be paid on diagnosis of patients exhibiting inclusion bodies in kidney cells, and it is important to confirm their past and present illnesses, and medication history as well as to measure the GLA activity and lyso-Gb3 level.

**Implication for health policy/practice/research/medical education:**

Three male patients are described in this case series, presenting with proteinuria and membranous cytoplasmic bodies (MCBs) in podocytes in kidney biopsies. MCBs in kidney cells are known to be the most important pathological finding for Fabry disease. But the results of assaying of α-galactosidase A (GLA) activity and measurement of the globotriaosylsphingosine concentration in plasma revealed that our patients did not have Fabry disease. Although the etiology of the MCBs has not been clarified yet, we should pay attention on diagnosis in cases exhibiting MCBs in kidney cells, and biochemical confirmation is necessary to avoid a pitfall in diagnosis of Fabry disease.

## 1. Background

Fabry disease (MIM 301500) is an X-linked genetic sphingolipidosis caused by a deficiency of α-galactosidase A (GLA, EC 3.2.1.22) (1,2). The enzymatic deficiency leads to impaired catabolism of glycosphingolipids, predominantly globotriaosylceramide (Gb3) and globotriaosylsphingosine (lyso-Gb3) (3-7). Although classically affected males have onset in childhood and exhibit systemic manifestations (classic variant) (1,2), there are other affected males which develop heart and/or kidney disease without the childhood symptoms (later-onset form) (2,8). Fabry females exhibit heterogeneous clinical presentations, ranging from asymptomatic to severe due to random X-chromosomal inactivation (1,2,9). Recent screening studies revealed that the incidence of Fabry disease was unexpectedly high (1 in 1250-8000 neonates), and many of the cases were later-onset variants (10-12). As most Fabry patients exhibit nonspecific manifestations of chronic kidney disease (CKD), the biopsy of kidney tissues is very important for finding them, and the most important pathological finding is the ultrastructural appearance of inclusions called lamellar inclusion bodies, zebra bodies, myeloid bodies or concentric electron dense bodies (1,2). Although such pathological changes are also recognized in other sphingolipidoses, they are limited to affected cells and tissues, ie, neurons and glia cells of the central nervous system in GM2 gangliosidosis (13), and they are not found in kidney cells. When such pathological inclusions are found in podocytes, and to a lesser extent, endothelial cells, tubular epithelial cells and mesangial cells, of the renal tissue, Fabry disease is strongly suspected (1,2,14).

However, less attention has been paid to the concept that there may be acquired lysosomal storage diseases presenting with inclusion bodies, ie, drug-induced lysosomal disease (15). Cationic amphiphilic drugs including chloroquine, hydroxychloroquine, chlorphentermine, chlorcyclizine, imipramine, chlorimipramine, and gentamycin have been reported to give pathological findings similar to those of Fabry disease (15-20). Furthermore, amiodarone, an antiarrhythmic agent, has attracted interest as a drug that causes chronic toxicity associated with intracellular inclusion bodies in various cells and tissues (21-23). These drugs inhibit lysosomal phospholipases, which catabolize phospholipids, and cause non-inherited phospholipidosis (15). Thus, the appearance of inclusion bodies in kidney cells can be related to treatment with these drugs that exhibit their own actions but cause similar pathologic changes, and therefore careful attention is required for differential diagnosis of Fabry disease. In this case series, we clinically and biochemically examined three male patients, who exhibited proteinuria and were suspected of having Fabry disease from the results of pathological examination, to avoid a wrong diagnosis.

## 2. Case series presentation

### 2.1. Patients

**Figure 1:**
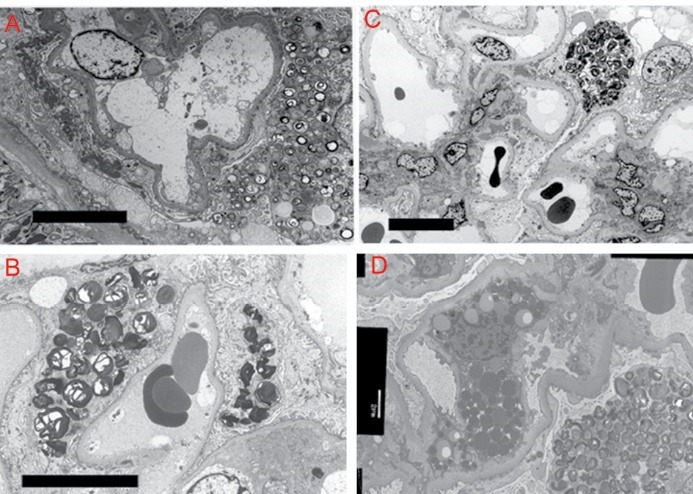
Electron microscopic findings in the biopsied kidney tissues from cases 1, 2, and 3, and a Fabry patient. (A) Case 1 (black scale bar: 10 μm), (B) Case 2 (black scale bar: 10μm), (C) Case 3 (black scale bar: 10 μm), and (D) a later-onset Fabry male harboring the M296I amino acid substitution, who developed proteinuria and left ventricular hypertrophy (white scale bar: 2 μm).

A summary of the patients examined in this study (cases 1, 2, and 3) is presented in Table 1. The patients exhibited proteinuria, and thus renal biopsies were performed for them. Electron microscopic examination revealed numerous inclusion bodies in podocytes (Figure 1A-C). The pathological findings mimicked those in a later-onset Fabry patient (Figure 1D). From the pathological findings, the pathologists who examined them diagnosed or strongly suspected Fabry disease.

**Table 1:** Major clinical manifestations and biochemical findings in the patients

**Subject**	**Age** **(y)**	**Sex**	**Major manifestations**	**Urinary protein (g/g creatinine)**	**Serum creatinine** ^a^ **(mg/100 mL)**	**GLA activity** ^b^ **(nmol/h/mL)**	**Lyso-Gb3** ^c^ **(nmol/L)**
Case 1	48	M	Proteinuria, LVH, apoplexy, and angiokeratoma	2.5	0.78	5.5	< 2
Case 2	72	M	Proteinuria	2.5	6.26	12.2	< 2
Case 3	33	M	Proteinuria	1.5	NA	7.9	< 2

Abbreviations: LVH, left ventricular hypertrophy; NA, not available. ^a^Normal range: 0.6-1.1 mg/100 mL;^b^Normal range: 3.9-13.1 nmol/h/mL; ^c^Normal range: < 2 nmol/L(6).

### 2.2. Measurement of plasma GLA activity and lyso-Gb3 concentrations

GLA activity in plasma was measured fluorometrically with 4-methylumbelliferyl α-D-galactopyranoside (Calbiochem, La Jolla, CA) as a substrate in the presence of N-acetyl-D-galactosamine (Sigma-Aldrich, St. Louis, MO) to specifically inhibit N-acetylgalactosaminidase in samples, as described previously (24). Plasma lyso-Gb3 was measured by means of high-performance liquid chromatography (HPLC), followed by fluorescence detection, as described previously (6).

### 2.3 Ethics statement

The research followed the tenets of the Declaration of Helsinki. Written informed consent for kidney biopsies followed by analysis for diagnosis were obtained from all the patients, although those for genetic analysis could not be obtained. The biochemical studies involving human samples were approved by the Ethics Committee of Meiji Pharmaceutical University.

### 2.4. Statistical analysis

To determine the normal range of plasma GLA activity, the mean value and the standard deviation (SD) were calculated using Excel 2013 (Microsoft, Rodmond, WA) on the basis of data from 30 healthy subjects, and it was determined to be mean±2SD. The GLA activity levels of the patients were examined whether they were out of the normal range or not.

### 2.5. Clinical data

### 2.5.1. Case 1

A 48-year-old male. When he was 10 years old, he presented with proteinuria and was diagnosed as having nephritis. In childhood, he suffered from left hemiplegia of unknown etiology. At 45 years, he developed arrhythmia and electrocardiographic examination revealed a complete right bundle branch block. At 47 years, hyperlipidemia, proteinuria, and microhematuria were found. His serum creatinine, total protein, and albumin levels were normal. He was treated with pitavastatin calcium and prednisolone, and underwent kidney biopsies. A light microscopic examination revealed segmental glomerular sclerosis and moderately increased mesangial cells. No apparent vacuolation was found in the kidney cells. Electron microscopic examination revealed numerous lamellar inclusion bodies in podocytes. But they were not detected in other kidney cells.

### 2.5.2. Case 2

A 72-year-old male. There was a past history of pancytopenia, proteinuria, and prostatic cancer. At age 68, due to recurrent fever, he was treated with clarithromycin and loxoprofen. Thereafter his renal function rapidly decreased over 2 months and the first renal biopsy was performed. Pathological examination revealed minor glomerular abnormalities, suggesting interstitial nephritis. Although urinary Bence-Jones protein (kappa) and monoclonal protein (IgG-kappa) were detected in this patient, drug-induced interstitial nephritis was also suspected because the drug lymphocyte stimulation tests were positive for both clarithromycin and loxoprofen. Bone marrow aspiration did not reveal any increase in plasma cells. He was treated with prednisolone and his renal functions improved. However, thereafter his proteinuria got worse and the serum creatinine level increased again, and a second renal biopsy was performed. Light microscopic examination revealed cytoplasmic vacuoles in podocytes but not in other kidney cells. No increase in mesangial cells and development of mesangium was found. There was no glomerulosclerotic change, although mild lymphocyte infiltration was found. Electron microscopic examination revealed numerous myeloid bodies in podocytes but not in other kidney cells.

### 2.5.3. Case 3

A 33-year-old male. At age 19 years, he suffered from idiopathic thrombocytopenic purpura and thus was treated with prednisolone. At age 23 years, he developed osteonecrosis of the femoral head and thus artificial joint replacement was performed. Due to persistent pain, he has taken loxoprofen until present. When he was 32 years old, proteinuria was found and a renal biopsy was performed. Antinuclear, anti-double stranded DNA, and anti-single stranded DNA antibodies were all positive. The serum C3 and C4 levels, and serum complement activity (CH50) were all within normal range. From the data, he was thought to have lupus nephritis. A renal biopsy was conducted, and light microscopic examination revealed moderate glomerulosclerosis and increased mesangial cells. Electron microscopic examination revealed numerous myeloid bodies in podocytes but not in other kidney cells. Immunofluorescence microscopy disclosed strong staining for IgG (subclass: IgG1>IgG4) and C3, and moderate staining for IgA and C1q in the area of the basement membrane and mesangium.

### 2.6. Plasma GLA activity and lyso-Gb3 concentration

From the pathological findings for renal biopsies (Figure 1), the patients were strongly suspected of having Fabry disease and thus their plasma GLA activities were measured. However, all the patients had normal GLA activity levels (5.5-12.2 nmol/h/mL, normal range; 3.9-13.1 nmol/h/mL) (Table 1). As they were male subjects with normal GLA activity, it was concluded that they do not to have Fabry disease.

The plasma lyso-Gb3 level, which is known to be a good biomarker of Fabry disease, was also measured. The results revealed that their lyso-Gb3 levels were within normal levels (<2 nmol/L) (Table 1), which supported the results of the GLA assay.

## 3. Discussion

Since high-risk screening studies have revealed many Fabry patients exhibiting manifestations of CKD (25-31), and enzyme replacement therapy with recombinant GLAs is available, it is becoming more and more important to make an early and correct diagnosis of Fabry disease (1,2). The existence of inclusion bodies in kidney cells constitutes strong evidence of Fabry disease. However, we should take care not to forget other possible causes that give pathological changes similar to those of Fabry disease. It has been reported that cationic amphiphilic drugs, ie, chloroquine, gentamycin and amiodarone, and their derivatives, cause accumulation of phospholipids in lysosomes and formation of lamellated lipid inclusions (15-32). Cationic amphiphilic drugs are compounds containing an aromatic or aliphatic ring and a hydrophilic region, being positively charged at physiologic pH. These drugs are potent inhibitors of lysosomal phospholipases, which leads to deposition of phospholipids in lysosomes. Although the detailed mechanism has not been clarified yet, these drugs bind to the hydrophilic moieties of phospholipids and probably form complexes not digestible by lysosomal phospholipases (32). Such pathological findings cannot be morphologically distinguished from those of Fabry disease. Amiodarone is also known to cause corneal opacities, one of the symptoms characteristic of Fabry disease (33).

As regards to cases 1 to 3, there is no history of cationic amphiphilic drugs (chloroquine, gentamycin and amiodarone, and their derivatives) having been prescribed for them. Unfortunately, samples for analyzing the substrates accumulated in lysosomes were not available, and the etiology of the inclusion bodies in these cases has not been elucidated yet. Cases 1, 2, and 3 suffered from hyperlipidemia, myeloma, and lupus nephritis, respectively, and case 1 had a medication history of pitavastatin calcium and prednisolone, case 2 also took clarithromycin, loxoprofen and prednisolone, and case 3 took loxoprofen and prednisolone too. There has been a report that electron dense deposits were found in the subintimal area of an arteriole in a patient with lupus nephritis (34). We could not find any evidence that our cases’ illnesses or medication histories are directly associated with lamellar inclusion bodies in podocytes. However, there may be unidentified drugs other than the cationic amphiphilic drugs or disorders that accelerate the formation of lipid complexes, and their combination may lead to nondigestible inclusion bodies mimicking those found in Fabry patients. Further study is required.

## 4. Conclusions

In summary, the existence of inclusion bodies is an important sign when considering Fabry disease but great care should be taken not to make the wrong diagnosis, and it is important to examine the past and present illnesses, and the medication history, as well as to measure the GLA activity and lyso-Gb3 level.

## 5. Limitations of the study

There are limitations to this study. We could not elucidate the cause of the intracellular inclusion bodies found in the biopsied kidney tissues from the patients. It will be necessary to biochemically analyze the substrates accumulated in kidney tissues when such cases are found in the future. Identification of the accumulated substrates will provide us with a lot of information to solve the problem.

## Authors’ contribution

HS designed and managed the research, and wrote the paper. TaTs, ToTa, and TaTo performed the biochemical analysis, and NT, DM, SW and YA performed the clinical analysis of the 3 male patients examined here and the Fabry patient.

## Conflicts of interest

The authors declare no conflict of interests.

## Funding/Support

This work was supported by the Program for the Promotion of Fundamental Studies in Health Sciences of the National Institute of Biomedical Innovation (ID: 09-15, HS); and the Program for Research on Intractable Disease of Health and Labor Science Research (HS).
